# The prevalence of exposure to domestic violence and the factors associated with co-occurrence of psychological and physical violence exposure: a sample from primary care patients

**DOI:** 10.1186/1471-2458-11-621

**Published:** 2011-08-04

**Authors:** Polona Selic, Katja Pesjak, Janko Kersnik

**Affiliations:** 1 Department of Family Medicine, Faculty of Medicine, University of Ljubljana, Poljanski nasip 58, Ljubljana, Slovenia

**Keywords:** Domestic violence, Physical Violence, Psychological Violence, Violence Prevalence, Risk Factors, Primary Care

## Abstract

**Background:**

Since many health problems are associated with abuse and neglect at all ages, domestic violence victims may be considered as a group of primary care patients in need of special attention.

**Methods:**

The aim of this multi-centre study was to assess the prevalence of domestic violence in primary care patients, and to identify those factors which influence the co-occurrence of psychological and physical violence exposure and their consequences (physical, sexual and reproductive and psychological) as obtained from medical records.

A study was carried out in 28 family practices in Slovenia in 2009. Twenty-eight family physicians approached every fifth family practice attendee, regardless of gender, to be interviewed about their exposure to domestic violence and asked to specify the perpetrator and the frequency. Out of 840 patients asked, 829 individuals, 61.0% women (n = 506) and 39.0% men (n = 323) were assessed (98.7% response rate). They represented a randomised sample of general practice attendees, aged 18 years and above, who had visited their physician for health problems and who were given a physical examination. Visits for administrative purposes were excluded.

Multivariate binary logistic regression analysis was used to determine the factors associated with exposure to both psychological and physical violence.

**Results:**

Of 829 patients, 15.3% reported some type of domestic violence experienced during the previous five years; 5.9% reported physical and 9.4% psychological violence; of these 19.2% of men and 80.8% of women had been exposed to psychological violence, while 22.4% of men and 77.6% of women had been exposed to physical violence. The domestic violence victims were mostly women (p < 0.001) aged up to 35 years (p = 0.001). Exposure to psychological violence was more prevalent than exposure to physical violence. Of the women, 20.0% were exposed to either type of violence, compared to 8.0% of male participants, who reported they were rarely exposed to physical violence, while women reported often or constant exposure to physical violence. Their partners were mostly the perpetrators of domestic violence towards women, while amongst men the perpetrators were mostly other family members.

In univariate analysis female gender was shown to be a risk factor for domestic violence exposure. Regression modelling, explaining 40% of the variance, extracted two factors associated with psychological and physical violence exposure: the abuse of alcohol in the patient (OR 4.7; 95% CI 1.54-14.45) and their unemployment (OR 13.3; 95% CI 1.53-116.45).

**Conclusions:**

As far as the study design permits, the identified factors associated with both psychological and physical violence exposure could serve as determinants to raise family physicians' awareness when exploring the prevalence of domestic violence. The results of previous research, showing at least 15% prevalence of exposure to domestic violence among primary care patients in Slovenia, and the female gender as a risk factor, were confirmed.

## Background

Domestic violence is a common and worrying social phenomenon. Ronan et al [1] stated that according to law enforcement statistics, domestic violence is the most frequent criminal activity in the USA. The victims are predominantly women and children [1,2]; men are less often exposed to violent behaviour within families. The data show that one out of seven women has experienced domestic violence and that 20-40% of women will become victims at least once in their lives [3,4]. According to a European Women's Lobby survey report, every fourth or fifth woman in the European Union has experienced violence from her partner [5]. The Council of Europe's findings are very similar, indicating that at least once in adulthood, between one fifth and one quarter of women experience domestic violence. In addition to physical violence, more than 10% of women have experienced sexual violence [6].

There are at least two distinct types of intimate partner violence (IPV) to be taken into consideration, i.e. common couple violence and intimate terrorism; the latter was considered to be the domain of men as perpetrators [7]. Johnson described several types of violence based on the dyadic control context of the violence, i.e. intimate terrorism, violent resistance, situational couple violence and mutual violent control [8]; these distinctions were mostly used in later research in this field. The population-based studies showed that between 25% and 50% of victims of IPV in a given year were men; female-perpetrated violence accounted for 40% of all cases reported during that time period [9,10]. A study by Hines and Douglas [11] was the first to provide a systematic, quantitative description of the IPV experiences of a large sample of men who sought help for IPV victimization. By comparing the sample of men who reported IPV, and sought help, with a community sample of men, the authors were able to gain a better understanding of the IPV experiences of both groups of men. The study showed the existence of male victims of female-perpetrated intimate terrorism (IT). These men sustained very high rates and frequencies of psychological, sexual, and physical IPV, injuries and controlling behaviours, congruent with the pattern of Johnson's [7] conceptualization of IT. Even though the male help-seekers had high rates of perpetrating IPV themselves, their rates were similar to or lower than those found in shelter samples of battered women, and their violent behaviour conformed to Johnson's conceptualization of violent resistance [12]. By these findings, Hines and Douglas [11] disproved Johnson's [7,8] assertion that IT is committed almost exclusively by men and violent resistance is committed almost exclusively by women.

In Slovenia, prior to the adoption of the Law on the Prevention of Domestic Violence, the only official data on domestic violence was collected by the police; however, the police only recorded data on reported crimes. According to these records the number of victims of domestic crime grew by 95% in the period 2000-2007, using 2000 as a baseline year. Applying these figures, in 2007 the police dealt with more than 2,700 victims of domestic violence in a country with only about two million inhabitants [13].

Many health problems are associated with abuse and neglect at all ages [1,3,4,14,15]. Murder is the most tragic outcome of domestic violence [16,17], but besides femicide, 60% of female domestic violence victims suffer direct (i.e. injuries: cuts, bruises, fractures) and indirect (e.g. gastrointestinal disorders, chronic pain, gynaecological disorders) health consequences of the abuse. From the psychological aspect, these women are 4-6 times more likely to suffer from depression than women who have not been exposed to domestic violence [15].

The data do not provide sufficient evidence to support intimate partner violence screening in health care settings [18]; whether intimate partner violence screening reduces violence or improves health outcomes for women has not yet been proven [18]. Some authors have proposed that violence screening should be part of general case history taking during consultation [19]. Patients in primary care would support a policy on screening for violence, especially when they are not comfortable enough to disclose the abuse on their own initiative [15,20]. On the other hand, a randomized controlled trial conducted in 11 emergency departments, 12 family practices, and 3 obstetrics/gynaecology clinics in Canada (totalling 6743 female patients aged 18-64 years) did not provide sufficient evidence to support domestic violence screening in health care settings [18], but suggested an evaluation of services for women, after identification of domestic violence, as a priority.

Qualitative studies of abused women have shown that the physician's interest in their exposure to domestic violence reduced the feelings of isolation often experienced in violence at home [14,20,21]. According to the literature in different cultural contexts, physicians do not ask patients about exposure to domestic violence during routine practice work. There are data on the implementation of some screening methods in 15-30% of patients [4,22]; however, findings emerging from different countries and cultures should not be generalised, aside from the impression that neither screening nor domestic violence case findings are generally used in primary care. As was clearly stated by Chen [23], culturally appropriate protocols are needed in primary care settings for prevention and intervention in relation to women at risk of domestic violence.

In Slovenia, there is a lack of data on the prevalence of domestic violence in the general population. In primary care the first study, in 2006, surveyed 27 family practices, including 1,103 patients, and showed that 40(3.6%) victims were men and 152(13.8%) were women. The authors analysed the patients' disclosure of physical and/or psychological domestic violence. Out of 1103 patients, 141(12.8%) individuals admitted that they had experienced both physical and psychological violence; 65(5.9%) patients reported that they had been victims of physical violence in the family; 120(10.9%) patients said that they had been victims of psychological violence; while 777(70.4%) individuals did not report any form of domestic violence [24]. In 2007, in another survey on the prevalence of domestic violence in primary care attendees in Slovenia, 25 General Practitioners (GPs) interviewed 797 consecutive patients who visited their surgeries; 295(37.0%) men and 502(63.0%) women. The survey [25] addressed the prevalence of domestic violence, the perpetrators, and the readiness of domestic violence victims to seek help. Of the sample, 97(12.2%) individuals (21(7.1%) men and 76(15.1%) women) reported being a victim of physical violence in the previous five years. Another 131(29%) patients, (47(15.9%) men and 84(36.7%) women) were victims of psychological violence within the family in the previous five years. A total of 85(10.7%) of those interviewed experienced both types of violence (12(4.1%) men and 73(14.5%) women) while 553(69.4%) patients (238(80.7%) men and 315(62.7%) women) did not report any kind of violent experience in their families.

Despite the high prevalence of domestic violence, and the proven harmful consequences on health, there is still no consensus on prevention strategies for domestic violence in family medicine or in Slovenia in general. The majority of studies identify two main reasons for the insufficient recognition of domestic violence victims; time limitation in primary care practices, and lack of professional knowledge. Physicians are not well informed about available recognition strategies and documenting methods, they do not feel competent to assess victims, and often they do not know the best means of intervention, or about existing institutions that work with victims of violent behaviour [2,3,26].

The aforementioned study in Slovenia in 2006 [24] showed that in one fifth of cases GPs did not do anything when patients asked for help in cases of domestic violence. Physicians suggested secondary care treatment to about a quarter of the victims, and they tried to discuss the problem with two-fifths of those seeking help.

As the majority of studies deal with domestic violence victims on the basis of population, the aim of this study was to determine the prevalence of domestic violence, and to identify the perpetrators and the determinants of exposure to psychological and physical violence in family practice patients, so that GPs are more able to detect them amongst the large numbers of patients in their practices.

## Methods

### Participants

In a multi-centre study, 28 family physicians from 28 family practices screened every fifth family practice attendee for domestic violence, starting on January 15, 2009 and ending on February 15, 2009. The participating family medicine practices were selected from both urban and rural settings, and served populations with diverse socio-economic and ethnic characteristics; the diversity and geographical representativeness of family care settings followed the study design described by Svab et al [27]. A random sample of general practice attendees regardless of gender, aged 18 years and above, who had visited their GP for health problems, and who were given a physical examination, were included in the study. Visits for administrative purposes, e.g. chronic patients coming for prescriptions and patients requiring sick leave forms, were excluded. No-one was accompanied by another person. The eligibility criteria were age, purpose of visit (health problems), and their willingness to participate. Each participating GP assessed 30 patients. The domestic violence exposure questionnaire (see Additional File 1) was administered after the examination and consultation about the health problem that was the reason for the attendance. Patients were invited to participate and told that it was not obligatory. Out of 840 invited patients, 829 were assessed (98.7% response rate). The 11(1.3%) people who did not want to discuss domestic violence did not disclose their motivation.

The National Medical Ethics Committee of the Republic of Slovenia approved the protocol of the study.

### Procedure

Many experts in the field of violence recognition have identified that a direct approach to violence screening is the most effective [14,28]. Inspired by this, although considering that there is insufficient evidence to support domestic violence screening in health care settings [18], in our study eligible patients were asked to answer questions about their exposure to psychological or/and physical violence, and to state the perpetrator and the frequency of exposure, with the aim of assessing the prevalence, perpetration and victimization of domestic violence in primary care attendees. The physicians specifically asked about the presence of violent behaviour in the family (i.e. *In the past five years, have you ever been beaten, slapped, kicked or in any other way exposed to physical violence at home?*), because this increases the likelihood of the victims' disclosure, as reported by other researchers in the field [14,29,30]. A question about coerced sexual intercourse followed (i.e. *Have you been in the last five years forced into sexual intercourse or any unwanted sexual behaviour?*). Due to the patients' negative response to this question (i.e. not even one patient answered "yes"), sexual violence is not presented as a special type of domestic violence in this study.

If the patient responded to the question about physical violence positively, they were then asked about the perpetrator (i.e. partner, parent, child, other family member) and the frequency [i.e. rarely (up to twice a year); occasionally (up to once a month); often (up to once a week) and constantly (more than once a week)] of the physical violence, with an additional question about coerced sexual intercourse.

Psychological violence was screened for by asking *In the past five years*, *have you been humiliated, subjected to threats, insult or intimidation, or in any way emotionally affected within the family? *If they answered yes, the patients were asked about the perpetrator and the frequency of the violence, as in the case of physical violence.

The second part of the survey was addressed to the GPs themselves; the questions related to the factors shown to be associated with exposure to domestic violence in previous Slovenian studies in primary care [24,25], and other generally accepted risk factors (Heise and Garcia-Moreno [31]). Further information on the participating patients was gathered by auditing their medical records, including data on the patients' wider life context. Two categories of data were abstracted from the medical records for each patient for the previous five years (2004-2008): firstly, the factors that are known to be associated with domestic violence [31] i.e. alcohol abuse; adult onset of depression; personality disorders; low education level (see Additional File 1); low income; unemployment in patient; past experience of violence, prior to screening period 2004-2008; conflict in intimate partner relationship; and male dominance in the family as a hardship, which were already discussed with the GP and marked in the medical record, and secondly, the impact of domestic violence on the patient's health [31] (physical consequences, sexual and reproductive consequences, psychological and behavioural consequences).

### Measures

A Domestic Violence Exposure Questionnaire, mostly derived from the work of Heise and Garcia-Moreno [31], was constructed and tested in previous studies in Slovenian primary care [24,25] (see Additional File 1). It consisted of questions about gender, age, number of children, marital status, number of divorces, residency, and exposure to violence (psychological and physical, including coerced sexual intercourse), frequency of exposure to violence, and the perpetrator of the violence. The physicians analyzed the patients' medical records and abstracted factors on both the personal level (i.e. alcohol abuse; adult onset of depression; personality disorders; low education level; low income; and unemployment in patient) and on a relationship level (i.e. past experience of violence (prior to screening period) reported by patient and marked in the medical records; conflict in intimate partner relationship, reported by patient and marked by physician; and male dominance in the family as a hardship, already discussed with the patient and marked by physician). The consequences of exposure to domestic violence were also listed and were later categorized into three groups: physical (i.e. bone fractures and skin wounds, bruises and abrasions, abdominal and chest injuries, head and eye injuries, fibromyalgia, chronic pain syndromes, poor general physical functioning) sexual and reproductive (i.e. sexual dysfunction, sexually transmitted infections, pelvic infections, infertility, complications of pregnancy/abortion, unplanned pregnancy and pregnancy termination), and psychological (i.e. low self-esteem, feelings of shame and guilt, phobias and panic disorders, post-traumatic stress disorder, eating and sleep disorders, depression and anxiety, suicide and self harm, psychosomatic disorders, smoking, alcohol and drug abuse, physical inactivity, risky sexual behaviours).

### Data Analysis

Sample data was presented by frequencies and percentages. The chi-square test was used to calculate the domestic violence exposure by demographic characteristics. Multivariate binary logistic regression analysis was used to determine the factors associated with exposure to both psychological and physical violence; the modelling included all the variables from the questionnaire (see Additional File 1). The calculation included the chi-square, odds ratio (OR), 95% confidence interval (95% CI) and P value. Statistical analysis was performed with SPSS 15.0 software. P < 0.05 was marked as statistically significant.

## Results

### Demographic Characteristics of Participants

From 840 patients invited, 829 - 61.0% women (n = 506) and 39.0% men (n = 323) - participated (98.7% response rate), of whom the majority (702, 84.7%) had not been exposed to psychological or physical violence within the family, including coerced sex, during the previous five years.

The sample demographic characteristics are presented in Table 1.

**Table 1:** Demographic Characteristics of the Respondents

Characteristic	All participants		Domestic violence Exposure				p-value	Psychol. violence		Psychol. and Physic. violence	
	
			no		yes						
								
	(n = 829)	%	(n = 702)	%	(n = 127)	%		(n = 78)	%	(n = 49)	%
**Gender**							< 0.001				
male	323	39.0	297	42.3	26	20.5		15	19.2	11	22.4
female	506	61.0	405	57.7	101	79.5		63	80.8	38	77.6
**Age (in years)**							0.001				
18-35	149	18.0	112	16.0	37	29.1		22	28.2	15	30.6
36-49	230	27.7	194	27.6	36	28.3		21	26.9	15	30.6
50-64	230	27.7	197	28.1	33	26.0		23	29.5	10	20.4
65 or above	220	26.5	199	28.3	21	16.5		12	15.4	9	18.4
**Marital status**							0.208				
single	103	12.4	85	12.1	81	63.8		9	11.5	9	18.4
in partnership	582	70.2	501	71.4	18	14.2		53	67.9	28	57.1
divorced	144	17.4	116	16.5	28	22.0		16	20.5	12	24.5
**Residency**							0.213				
rural	297	35.8	258	36.8	39	30.7		24	30.8	15	30.6
suburbs	128	15.4	111	15.8	17	13.4		6	7.7	11	22.4
urban	404	48.7	333	47.4	71	55.9		48	61.5	23	46.9
**Number of divorces**							< 0.001				
none	685	82.6	588	83.8	97	76.4		62	79.5	35	71.4
one	131	15.8	109	15.5	22	17.3		12	15.4	10	20.4
two	13	1.6	5	0.7	8	6.3		4	5.1	4	8.2
**Number of children**							0.735				
none	157	18.9	129	18.4	28	22.0		17	21.8	11	22.4
one	218	26.3	185	26.4	33	26.0		24	30.8	9	18.4
two	303	36.6	257	36.6	46	36.2		29	37.2	17	34.7
three or more	151	18.2	131	18.7	20	15.7		8	10.3	12	24.5

The sample consisted of 323(39.0%) men and 506(61.0%) women. The proportion of women in our sample (Table 1) was higher (61.0 *vs*. 54.8%), than the representative sample of Slovenian general practice attendees described by Svab et al [27].

Of these, 15(19.2%) males and 63(80.8%) females had been exposed to psychological violence, while 11(22.4%) men and 38(77.6%) women had been exposed to physical violence. The domestic violence victims were mostly women (p < 0.001) and aged up to 35 years (p = 0.001).

The victims of physical violence were all exposed to psychological violence. They were mostly living in intimate partnerships, including both marriage and common-law partnerships; the others were divorced or single. The victims of physical violence were mostly living in intimate partnerships, and the others were either single or divorced at the time of data collection. Most of the people exposed to either type of violence had not experienced divorce; however a greater percentage of those who were divorced also experienced domestic violence.

### Domestic Violence Exposure: Types and Perpetrators

The frequency of co-occurring physical and psychological domestic violence exposure in male and female participants and its perpetrators is presented in Table 2.

**Table 2:** Domestic Violence Exposure: Types and Perpetrators

Domestic violence types	Within all participants (n = 829)		Reported cases of violence (n = 127)		**Psychol**. violence (n = 78)		**Psychol. and Physic**. violence (n = 49)	
	
	Male (n = 323)	Female (n = 506)	Male (n = 26)	Female (n = 101)	Male (n = 15)	Female (n = 63)	Male (n = 11)	Female (n = 38)
**Domestic violence exposure**				p = 0.057		p = 0.465		p = 0.002
rarely	3.7	4.3	46.2	21.8	33.3	28.6	63.6	10.5
occasionally	1.2	6.7	15.4	33.7	20.0	41.3	9.1	21.1
often	0.9	3.8	11.5	18.8	20.0	12.7	0.0	28.9
constantly	2.2	5.1	26.9	25.7	26.7	17.5	27.3	39.5
**Domestic violence perpetrator**				p = 0.008		p = 0.542		p = 0.005
partner	2.2	12.1	26.9	60.4	40.0	55.6	9.1	68.4
partner and other family member	1.2	1.2	15.4	5.9	13.3	6.3	18.2	5.3
other family member	4.6	6.7	57.7	33.7	46.7	38.1	72.7	26.3

All the participants who were exposed to physical violence were also exposed to psychological violence. Female participants were exposed to both types of violence in greater proportion; the exposure was often or constant (p = 0.002) and perpetrators were intimate partners (p = 0.005), while male participants reported rare exposure to co-occurring psychological and physical violence, and the perpetrators were mostly other family members. There were no significant differences according to gender, frequency or perpetrators in psychological violence exposure.

### Factors Associated with Exposure to Psychological and Physical Violence: A Multivariate Regression Model

Table 3 shows the characteristics of victims exposed to both psychological and physical violence within a family (χ^2 ^= 44.162, df = 22, p = 0.003). The sensitivity and specificity in the modelling process were 76.3% and 77.5% respectively.

**Table 3:** Factors Associated with Exposure to Psychological and Physical Violence: A Multivariate Regression Model

	Domestic violence (%)		**χ**^**2**^	OR with 95% CI	p-value
				
	Psychol. (n = 78)	Psychol. and Physic. (n = 49)			
Female gender	80.8	77.6	0.1	0.8 (0.24-2.75)	0.744
**Age **(in years)					
18-35	28.2	30.6		1.0	
36-49	26.9	30.6	0.1	0.8 (0.18-3.46)	0.765
50-64	29.5	20.4	0.1	0.8 (0.17-3.35)	0.708
65 or above	15.4	18.4	0.5	1.8 (0.33-9.61)	0.498
**Marital status**					
single	11.5	18.4		1.0	
in partnership	67.9	57.1	2.7	0.2 (0.05-1.32)	0.102
divorced	20.5	24.5	1.1	0.3 (0.04-2.76)	0.302
**Residency**					
rural	30.8	30.6		1.0	
suburbs	7.7	22.4	0.8	2.1 (0.40-10.92)	0.377
urban	61.5	46.9	0.3	0.7 (0.24-2.25)	0.590
**Number of divorces**					
none	79.5	71.4		1.0	
one	15.4	20.4	1.3	2.4 (0.55-10.10)	0.250
two	5.1	8.2	0.0	1.1 (0.10-12.84)	0.924
**Number of children**					
none	21.8	22.4		1.0	
one	30.8	18.4	0.2	0.7 (0.13-3.85)	0.698
two	37.2	34.7	0.0	1.2 (0.19-7.28)	0.864
three or more	10.3	24.5	0.5	2.1 (0.28-16.50)	0.464
Alcohol abuse in patient	15.4	44.9	7.4	4.7 (1.54-14.45)	0.007
Depression in patient	16.7	30.6	0.4	1.5 (0.45-5.20)	0.503
Low education in patient	17.9	36.7	0.6	0.6 (0.14-2.39)	0.454
Low income in the family	16.7	40.8	2.2	2.7 (0.73-9.71)	0.138
Smoking	16.7	22.4	0.8	0.5 (0.13-2.11)	0.367
Gastrointestinal disorder	12.8	26.5	1.0	2.0 (0.53-7.44)	0.312
Chronic pain	9.0	22.4	0.3	0.7 (0.15-2.88)	0.585
Unemployment	2.6	22.4	5.5	13.3 (1.53-116.45)	0.019

Nagelkerke R Square = 0.399

The results of the modelling procedure explained nearly 40% of the variance (Nagelkerke R Square = 0.399), with alcohol abuse in the patient (OR 4.7; 95% CI 1.54-14.45) and their unemployment (OR 13.3; 95% CI 1.53-116.45) being the only obvious factors associated with both psychological and physical violence exposure. Low income, which was associated with exposure to both types of domestic violence, was close to the level of significance, as was living in an intimate partnership, which reduced the risk of psychological violence and physically violent behaviour. It follows that a single person was more at risk of being exposed to physical violence, with family members being the perpetrators. Apparently, living in intimate partnerships involved more quarrelling, shouting and insults.

Low income and a low level of education (vocational school or lower) were strongly correlated. Low income increased the likelihood, while a low level of education had the opposite effect, acting as a risk reducing factor. Although this seems illogical, it is due to the strong correlation between the two.

### The Factors Strongly Associated with Exposure to both Psychological and Physical Violence

There were 14 people with a history of unemployment (3 men (0.9%) and 11 women (2.2%)), and in 12 of these cases, psychological violence appeared together with physical violence. The percentage of cases in which exposure to both types of violence was identified was almost equal whether alcohol abuse was present in the patient or not. Alcohol abuse was shown to be in a stronger association with psychological and physical violence exposure than a history of unemployment due to its prevalence (n = 34).

**Figure 1:**
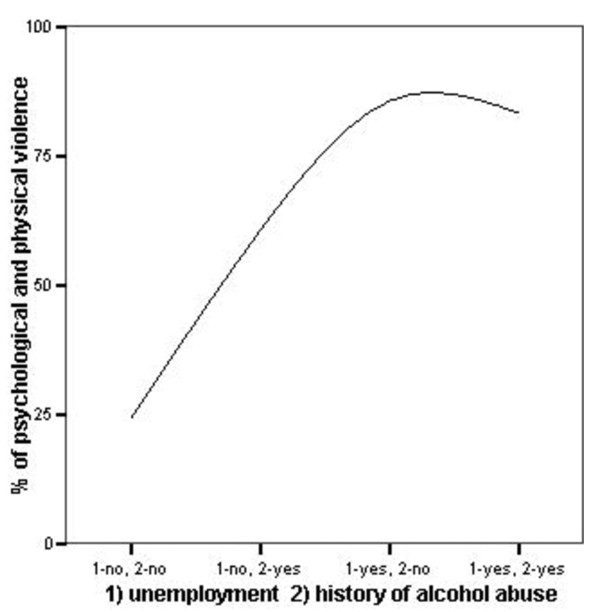
**The Factors Strongly Associated with Exposure to both Psychological and Physical Violence**. The percentage of psychological and physical violence cases versus psychological violence cases alone, according to: 1) unemployment 2) history of alcohol abuse.

Figure 1 shows the percentage of both types of violence cases versus psychological violence cases according to the factors strongly associated with both psychological and physical violence exposure, i.e. unemployment and alcohol abuse in the patient.

## Discussion

This study determined the prevalence of violence in family practice patients during the period from 2004-2008, and identified the perpetrators and the factors associated with co-occurring exposure to psychological and physical violence in primary care patients.

The results of a cross-sectional survey show that exposure to psychological domestic violence is more frequent than exposure to physical violence (Table 1). A significantly greater percentage of victims of violence in total are women (Table 1). In univariate analysis we were able to confirm that female gender was a greater risk for domestic violence exposure, but later in the second part of data analysis, when the co-occurrence of psychological and physical violence exposure was evaluated in the regression model, female gender was not shown to be a significant risk factor. However, a higher prevalence in women (Table 1) is consistent with the results of other studies [2,15,21]. Intimate partners were most often identified as the perpetrators of violence in the family (Table 2). Again, the data are consistent with findings on the prevalence of different kinds of violence from other authors [25,32-34], although it must be noted that our study was conducted in primary healthcare settings and should not be compared with those conducted in the general population. Garcia-Moreno et al [33] reported that the lifetime prevalence of physical or sexual, or both, partner violence in ten countries (Bangladesh, Brazil, Ethiopia, Japan, Namibia, Peru, Samoa, Serbia and Montenegro, Thailand, and the United Republic of Tanzania) varied from 15% to 71%; our study sample consisted of adult primary care attendees, so there are limitations to a comparison. Exposure to physical violence from an intimate partner, at any time in their lives, was reported in various studies by 22% of women in the U.S., 34% of women in Canada, 23% of women in Serbia and 15% of women in Japan [34]. The findings of the present study showed a lower prevalence of domestic violence than these, which may be due to the different study populations. The only extensive public opinion poll of domestic violence in Slovenia, conducted in 2005 from a representative sample of the adult population (1006 respondents), showed a 23.7% prevalence of any kind of domestic violence (24.2% male and 23.2% female) [35]. Among those who had personally experienced domestic violence, 73% of respondents said that it had happened in the family in which they grew up, while 38.6% reported that it was in the family in which they lived as adults. Some had experienced violence both in childhood and in their adult partnership. Significantly more women than men had personally experienced domestic violence as adults, which is concordant with our findings that the domestic violence victims were mostly women (p < 0.001; Table 1).

We did not find statistically significant differences in regard to the patients' living environment (rural, suburbs, urban; p = 0.213), although patients from urban areas reported a higher percentage of violent experiences in their families (Table 1), which is in accordance with some other studies [25,36]. No statistically significant correlation was shown between the number of the patients' children and domestic violence exposure. The results of this study are consistent with a study by Selic et al, 2008 [25].

Considering other demographic characteristics apart from gender, significant differences in age and the number of divorces were found between the groups (Table 1). Most cases of domestic violence were reported in women of a younger age (up to 35 years; p < 0.001; Table 1), while the fewest were reported in the age group 65 years or above. Being divorced once or twice was also identified as a factor associated with exposure to domestic violence, although there were only a few people who had experienced two divorces (n = 13); of these, 8(61.5%) reported domestic violence within the past five years. Although the authors tried to avoid seeing problems of violence only at the individual level by presenting the perpetrators and victims as an interactive relationship, the study design failed to address the real complexity of the interaction of factors within the family. Further research is needed to include factors such as family conflict, parental roles, parental neglect, the family background as a whole, approaches to discipline and guidance of children, and relationships between parents and children. In family medicine, the identification of dysfunctional interpersonal relationships and appropriate multilateral action are essential for the successful recognition and prevention of violence and the support of the victim and the family.

The main aim of this research was to determine those risk factors associated with co-occurring exposure to psychological and physical violence. The regression modelling explained nearly 40% of the variance, and extracted two factors, i.e. alcohol abuse and a history of unemployment in the victim (Table 3). Alcoholism and other substance abuse too often go unrecognized. As Slovenia is known for a high rate of alcohol dependence [37], we may assume that unemployment increases the risk of exposure to both psychological and physical violence in association with a history of alcohol abuse in the victim, which was identified by our study as the most important risk factor for co-occurring exposure to psychological and physical violence (Figure 1). Poverty is associated with unemployment [38] (Figure 1) and is linked to violence and abuse. Gender, for example, known as one of the main risk factors for domestic violence incidents, was not a significant determinant of exposure to both psychological and physical violence, since there were no differences between exposure to psychological violence alone and co-occurring psychological and physical violence.

Due to different subjective understanding of the criteria for psychological violence in victims, which were not explored in detail by the physicians, there could be relatively large differences in the estimated frequency and consequences of these incidents. As domestic violence may be considered an interpersonal phenomenon, we can conclude that future research should include data on the perpetrator as well as on the victim-perpetrator relationship, in order to be more accurate and explain a greater percentage of the variance.

It was originally the authors' intention to identify physical, sexual and psychological violence in a randomised sample of primary care patients, to determine the frequency and to identify the perpetrators. However, in spite of a structured interview procedure, the attending physicians did not identify a single case of coerced sexual intercourse. This may be due to their lack of training or a lack of motivation, as well as to the patient-family doctor interaction or feelings of shame in the patients. Therefore the first limitation to the result of this survey is missing data on sexual violence, as we are only able to present data on the prevalence of physical and psychological violence. Incomplete differentiation and understanding of domestic violence may lead to generalized and inappropriate actions on the ground, and to providing unsuitable assistance to the affected people.

The second limitation is that family doctors do not sufficiently distinguish between the different effects of domestic violence on men and women. In particular, the male-female interpersonal interaction within the vicious circle of abuse and violence is insufficiently examined; GPs, as well as the public, usually recognize women as victims of domestic violence [11,35]. All of the male victims in Hines and Douglas study [11] indicated that they had sought help in some form; it is obvious that training for members of the caring professions should include information about men's IPV victimization. Systematic research and education in this direction is also necessary in the field of family medicine.

It is an advantage that our findings are based on a randomised sample of family practice attendees in Slovenia, so the identified risk factors which are associated with co-occurring exposure to psychological and physical violence could serve as relatively valid guidance for family physicians. The present study confirmed the results of previous research by Selic et al [24,25] on the prevalence of exposure to domestic violence amongst primary care patients in Slovenia. For more effective identification of domestic violence victims, a different approach should be used (e.g. in-depth interviews with trained interviewers) and other means should be developed to encourage the victims of family violence to seek help on their own, or at least to be ready to disclose victimization when asked by health workers. Although the literature on family and intimate partner violence is extensive, few studies provide data on detection and management to guide clinicians [39]. It should be noted that while women are the most common victims of domestic violence, men of different age groups may also be victims. Better detection, not yet validated, would probably help them, and would also help to explain the multidimensional problem of domestic violence. It would shape clear action directives, expectations and demands. As stated by Nelson et al [39], studies of the effectiveness of treatment programs for abused victims, as well as for perpetrators, would provide much needed evidence that identification and intervention can lead to improved health outcomes.

## Conclusions

The two risk factors associated with exposure to both psychological and physical violence, i.e. the abuse of alcohol in the patient and the patient's unemployment, should be accepted as relatively valid guidance for family physicians while exploring the possibility of domestic violence exposure in patients. Since family medicine covers the adult population in Slovenia and the present study is the third on the prevalence of domestic violence amongst primary care patients here, an exposure rate of 15% or more should be addressed as a serious public health issue.

## List of Abbreviations

GPs: family medicine physicians/General Practitioners; IPV: intimate partner violence; IT: intimate terrorism; Physic. violence: Physical violence; Psychol. violence: Psychological violence.

## Competing interests

The authors declare that they have no competing interests.

## Authors' contributions

PS conceived the study, carried out the coordination, and drafted the manuscript. JK participated in the data collection and interpretation and helped to draft the manuscript. KP participated in the execution of the study and helped to draft the manuscript. All authors read and approved the final manuscript.

## Authors' information

PS: PhD Clinical Psychology, Senior Researcher and Assistant Professor at the Department of Family Medicine

JK: PhD Family Medicine, Professor, Head of the Research Division at the Department of Family Medicine,

KP: PhD Sociology, Junior Researcher

## Pre-publication history

The pre-publication history for this paper can be accessed here:

http://www.biomedcentral.com/1471-2458/11/621/prepub

## Supplementary Material

Additional file 1 **Domestic Violence Exposure in Primary Care Patients Summary Sheet/IZPOSTAVLJENOST NASILJU V DRUŽINI - ZBIRNI LIST (in Slovene)**. (Domestic Violence Exposure in Primary Care Patients Summary Sheet.doc) Click here for file
