# Supplementary material for: The prevalence of exposure to domestic violence and the factors associated with co-occurrence of psychological and physical violence exposure: a sample from primary care patients
Source: BMC Public Health. 2011 Aug 4;11:621. doi: 10.1186/1471-2458-11-621 (PMC3160996; doi:10.1186/1471-2458-11-621)
Supplement: Additional file 1 — Domestic Violence Exposure in Primary Care Patients Summary Sheet/IZPOSTAVLJENOST NASILJU V DRUŽINI - ZBIRNI LIST (in Slovene). (Domestic Violence Exposure in Primary Care Patients Summary Sheet.doc) [file 1471-2458-11-621-S1.DOC]

# Domestic Violence Exposure in Primary Care Patients Summary Sheet

1. Gender: Male Female
2. Age ________ years
3. Marital status

|  in partnership (married or living with partner) |
| --- |
|  single |
|  divorced |

1. Number of children ________
2. If divorced, number of divorces ____
3. Residency / Locality □ Rural

□ Suburban

□ Urban

1. In the past five years, have you ever been beaten, slapped, kicked or in any other way exposed to physical violence at home?

YES/NO

1. If YES: by whom?
2. If YES: How often? Tick below:

|  rarely (up to twice a year |
| --- |
|  occasionally (up to once a month) |
|  often (up to once a week) |
|  constantly (more than once a week) |

1. Have you been in the last 5 (five) years, forced into sexual intercourse or any unwanted sexual behaviour?

YES/NO

1. If YES: by whom?
2. If YES: How often? Tick below:

|  rarely (up to twice a year |
| --- |
|  occasionally (up to once a month) |
|  often (up to once a week) |
|  constantly (more than once a week) |

1. In the past five years, have you been humiliated, subjected to threats, insult or intimidation, or in any way emotionally affected within the family?

YES/NO

1. If YES: by whom?
2. If YES: How often? Mark below

|  rarely (up to twice a year |
| --- |
|  occasionally (up to once a month) |
|  often (up to once a week) |
|  constantly (more than once a week) |

1. If patient answered positively on more than one question about exposure to violence:

Which occurred first?

□ Physical violence

□ Sexual violence

□ Psychological violence

1. Circumstances which increase the possibility of violence: review patient’s medical records for the previous five years (2004-2008) and tick below - more answers are possible

|  Alcohol abuse  Who in the family (patient, partner, other family member)? |
| --- |
|  Adult onset of depression  Who in the family (patient, partner, other family member)? |
|  Personality disorders  Who in the family (patient, partner, other family member)? |
|  Low education level  Who in the family (patient, partner, other family member)? |
|  Low income |
|  Unemployment in patient |
|  Past experience of violence (prior to screening period 2004-2008) |
|  Conflict in intimate partner relationship |
|  Male dominance in the family as a hardship, already discussed |

1. Health consequences of domestic violence - review medical records and tick below - more answers are possible

PHYSICAL CHANGES / SIGNS

 Bone fractures and skin wounds

 Bruises and abrasions

 Abdominal and chest injuries

 Head and eye injuries

 Fibromyalgia

 Chronic pain syndromes

 Poor general physical functioning

SEXUAL AND REPRODUCTIVE CONSEQUENCES

 Sexual dysfunction

 Sexually transmitted infections

 Pelvic infections

 Infertility

 Complications of pregnancy / abortion

 Unplanned pregnancy and pregnancy termination

PSYCHOLOGICAL AND BEHAVIOURAL IMPLICATIONS

 Low self-esteem

 Feelings of shame and guilt

 Phobias and panic disorder

 Post-traumatic Stress Disorder

 Eating disorders and sleep disorders

 Depression and Anxiety

 Suicide and self-harm

 Psychosomatic disorder

 Smoking, alcohol and drug abuse

 Physical inactivity

 Risky sexual behaviours

**IZPOSTAVLJENOST NASILJU V DRUŽINI - ZBIRNI LIST**

1. Spol M Ž
2. Starost ________ let
3. Stan

|  živi s partnerjem/partnerko (poročen ali v zunaj-zakonski skupnosti) |
| --- |
|  samski |
|  ločen |

1. Število otrok ________
2. Če je oseba razvezan/a, navedite število razvez ____
3. Bivališče □ vaško

□ primestno

□ mestno

1. Ali ste bili v zadnjih 5 (petih) letih doma (v družini) tepeni, oklofutani, obrcani ali kakorkoli izpostavljeni telesnemu nasilju? DA NE
2. Če DA: S strani koga?
3. Če DA: Kako pogosto? označite s križcem

| |  Zelo redko (do 2 – krat letno) | | --- | |  Včasih (do 1 – krat mesečno) | |  Pogosto (do 1 – krat tedensko)   Neprestano (več kot enkrat tedensko) | |
| --- | --- | --- | --- |

1. Ali ste bili v zadnjih 5 (petih) letih prisiljeni v spolno občevanje ali kakršnokoli neželeno spolno vedenje? DA NE
2. Če DA: S strani koga?
3. Če DA: Kako pogosto? označite s križcem

| |  Zelo redko (do 2 – krat letno) | | --- | |  Včasih (do 1 – krat mesečno) | |  Pogosto (do 1 – krat tedensko)   Neprestano (več kot enkrat tedensko) | |
| --- | --- | --- | --- |

1. Ali ste bili v zadnjih 5 (petih) letih doma (v družini) ponižani, izpostavljeni grožnjam, žalitvam, zastraševanju ali kakorkoli čustveno prizadeti? DA NE
2. Če DA: S strani koga?
3. Če DA: Kako pogosto? označite s križcem

|  Zelo redko (do 2 – krat letno) |
| --- |
|  Včasih (do 1 – krat mesečno) |
|  Pogosto (do 1 – krat tedensko) |
|  Neprestano (več kot enkrat tedensko) |

1. Če bolnik/ca odgovori pritrdilno na več kot eno vprašanje o izpostavljenosti nasilju, vprašate:

Kaj se je pojavilo prej?

□ telesno nasilje

□ spolno nasilje

□ psihično nasilje

1. Okoliščine, ki povečujejo možnost nasilja – odgovor zdravnika po pregledu zdravstvenega kartona bolnika/ce za obdobje 2004-2008 označite s križcem – možnih je več odgovorov

|  Alkoholizem  Kdo v družini (bolnik/ca, partner, drugi družinski člani) |
| --- |
|  Depresivnost  Kdo v družini (bolnik/ca, partner, drugi družinski člani) |
|  Osebnostne motnje  Kdo v družini (bolnik/ca, partner, drugi družinski člani) |
|  Nizka izobrazba  Kdo v družini (bolnik/ca, partner, drugi družinski člani) |
|  Nizki dohodki |
|  Nezaposlenost bolnika/ce |
|  Izkušnja z nasiljem pred letom 2004 |
|  Konflikti v partnerskem odnosu |
|  Dominantnost moškega v družini |
|  Brezposelnost, težave z iskanjem zaposlitve |

1. Zdravstvene posledice nasilja v družini – odgovor zdravnika po pregledu zdravstvenega kartona -označite s križcem – možnih je več odgovorov

| TELESNE SPREMEMBE/ZNAKI   Zlomi in raztrganine   Modrice in odrgnine   Poškodbe trebuha in prsnega koša   Poškodbe glave in očesa   Fibromialgija   Kronični bolečinski sindromi   Slabše splošno fizično funkcioniranje  SPOLNE IN REPRODUKTIVNE POSLEDICE   Spolna disfunkcija   Spolno prenosljive okužbe   Pelvična vnetja   Neplodnost   Komplikacije nosečnosti / sp. splav   Neželena nosečnost in splav  PSIHOLOŠKE IN VEDENJSKE POSLEDICE   Nizko samospoštovanje   Občutki sramu in krivde   Fobije in panične motnje   Post-travmatska stresna motnja   Motnje hranjenja in motnje spanja   Depresija in anksioznost   Samomorilnost in samopoškodovanje   Psihosomatske motnje   Kajenje, zloraba alkohola in drog   Telesna nedejavnost   Tvegano spolno vedenje |
| --- |
